# Modulating the Gut Microbiota to Target Neuroinflammation, Cognition and Mood: A Systematic Review of Human Studies with Relevance to Fibromyalgia

**DOI:** 10.3390/nu17142261

**Published:** 2025-07-09

**Authors:** Gianna Dipalma, Grazia Marinelli, Laura Ferrante, Angela Di Noia, Claudio Carone, Valeria Colonna, Pierluigi Marotti, Francesco Inchingolo, Andrea Palermo, Gianluca Martino Tartaglia, Massimo Del Fabbro, Angelo Michele Inchingolo, Alessio Danilo Inchingolo

**Affiliations:** 1 Interdisciplinary Department of Medicine, University of Bari “Aldo Moro”, 70124 Bari, Italy; giannadipalma@tiscali.it (G.D.); graziamarinelli@live.it (G.M.); lauraferrante79@virgilio.it (L.F.); angeladinoia@libero.it (A.D.N.); claudio.carone@uniba.it (C.C.); valeria.colonna@uniba.it (V.C.); pierluigi.marotti@uniba.it (P.M.); angeloinchingolo@gmail.com (A.M.I.); ad.inchingolo@libero.it (A.D.I.); 2 Department of Experimental Medicine, University of Salento, 73100 Lecce, Italy; andrea.palermo@unisalento.it; 3 Department of Biomedical, Surgical and Dental Sciences, Milan University, 20122 Milan, Italy; gianluca.tartaglia@unimi.it (G.M.T.); massimo.delfabbro@unimi.it (M.D.F.); 4 Unit of Maxillo-Facial Surgery and Dentistry, Fondazione IRCCS Ca’ Granda Ospedale Maggiore Policlinico, 20122 Milan, Italy

**Keywords:** brain–gut interaction, microbiota, neuroinflammatory response, fibromyalgia, gut–brain axis, probiotics, prebiotics, neuroinflammation, cognitive dysfunction, pain management

## Abstract

Aim: This systematic review aims to evaluate the effectiveness of microbiota-modulating interventions (such as probiotics, prebiotics, and fecal microbiota transplantation) in reducing cognitive symptoms, pain, and neuroinflammation in human studies relevant to fibromyalgia (FM). The review will investigate the role of gut–brain axis modulation through these interventions and explore the potential therapeutic benefits for FM management. Materials and Methods: A comprehensive search was conducted in electronic databases including PubMed, Scopus, and the Cochrane Library for studies published from 1 January 2015 to 30 April 2025. Studies were eligible if they were randomized controlled trials (RCTs), pilot studies, or observational studies assessing the impact of microbiota-targeted interventions (probiotics, prebiotics, fecal microbiota transplantation) on cognitive function, pain, or neuroinflammation in patients with FM. Studies were excluded if they involved animal models, lacked relevant outcome measures, or were not peer-reviewed. Although only a subset of the included studies directly involved FM patients, all were selected for their relevance to symptom domains (e.g., pain, cognition, mood) and mechanisms (e.g., neuroinflammation, gut–brain axis dysfunction) that are central to FM. A total of 11 human studies were included in the final qualitative synthesis. Results: Preliminary findings from the included studies suggest that microbiota-targeted interventions, particularly probiotics and prebiotics, show promise in reducing cognitive symptoms, pain, and neuroinflammation in FM patients. Improvements in mood and quality of life were also reported, indicating potential benefits for overall well-being. However, heterogeneity in study designs, sample sizes, and outcome measures limit the ability to draw definitive conclusions. Conclusions: This systematic review highlights the potential of microbiota modulation as a therapeutic strategy for managing FM symptoms, particularly cognitive dysfunction and neuroinflammation.

## 1. Introduction

### 1.1. Clinical Context: Understanding Fibromyalgia and Its Symptomatology

Fibromyalgia (FM) is a chronic and complex disorder characterized primarily by widespread musculoskeletal pain, persistent fatigue, and heightened sensitivity to tactile stimuli. It affects approximately 2–4% of the general population, with a significantly higher prevalence in women [1,2,3,4,5]. Despite its high burden, FM remains one of the most poorly understood conditions within rheumatology and pain medicine [6,7,8,9,10]. Diagnosis is based on symptom criteria, including chronic pain, fatigue, unrefreshing sleep, cognitive difficulties (commonly termed “fibro fog”), and somatic symptoms such as irritable bowel syndrome (IBS), headaches, or bladder discomfort [11,12,13,14,15]. In addition to physical symptoms, FM is closely associated with a range of psychiatric comorbidities. A substantial proportion of patients suffer from anxiety, depression, post-traumatic stress disorder, and emotional dysregulation, all of which may exacerbate pain perception and functional disability [16,17,18,19,20]. These psychological dimensions not only complicate the clinical profile but also suggest a possible role for central nervous system (CNS) dysfunction in the pathogenesis of FM. Cognitive impairment in Fibromyalgia (FM), often described by patients as mental cloudiness, difficulty concentrating, and memory lapses (collectively referred to as “fibro fog”), represents a particularly disabling aspect of the disease [21,22,23,24,25]. These symptoms resemble those in chronic fatigue syndrome (CFS) and mild cognitive impairment (MCI), and may be linked to brain connectivity changes, neuroinflammation, or neurotransmitter imbalances.

Fatigue affects over 90% of FM patients and is often resistant to standard treatments. Gastrointestinal complaints such as bloating, constipation, diarrhea, or alternating bowel habits are also common. The frequent overlap between FM and IBS—a fellow functional somatic syndrome—suggests shared mechanisms, possibly involving gut–brain axis disruption [26,27,28,29,30,31].

### 1.2. Biological Rationale: The Gut–Brain Axis and Microbiota in FM

**Figure 1:**
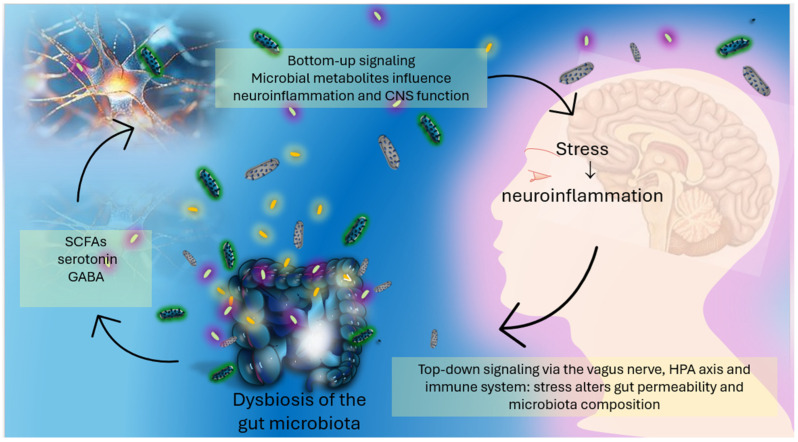
The gut–brain axis: bidirectional communication between the gut microbiota and the central nervous system. Microbial metabolites such as Short-Chain Fatty Acids (SCFAs), gamma-aminobutyric acid (GABA), and serotonin are produced in the gut and influence brain function and neuroinflammation via bottom-up signaling. Conversely, stress-related brain signals modulate gut permeability and microbiota composition through top-down pathways involving the vagus nerve, the hypothalamic–pituitary–adrenal (HPA) axis, and immune regulation. Dysbiosis can amplify this cycle, contributing to neuropsychiatric and pain-related symptoms.

Over the last decade, the concept of the gut–brain axis has gained considerable attention in the context of chronic pain and neuropsychiatric conditions. This bidirectional communication network between the gastrointestinal tract and the CNS involves neural, endocrine, and immune pathways and is modulated in part by the gut microbiota (Figure 1) [32,33,34,35,36,37,38].

Emerging evidence points to the gut microbiome as a critical regulator of systemic inflammation, neuroimmune signaling, stress response, and even behavior [39,40,41,42,43,44,45]. In FM, proposed pathophysiological mechanisms include central sensitization, HPA axis dysfunction, mitochondrial impairment, and low-grade inflammation [46,47,48,49,50,51,52]. More recently, neuroinflammation, defined as chronic, low-level activation of glial cells in the brain and spinal cord, has been recognized as a potential contributor to the amplified pain and cognitive symptoms seen in FM [53,54,55,56,57,58,59]. Such states may be triggered by microbial products (e.g., lipopolysaccharide) or altered gut permeability, often described as “leaky gut.”

**Figure 2:**
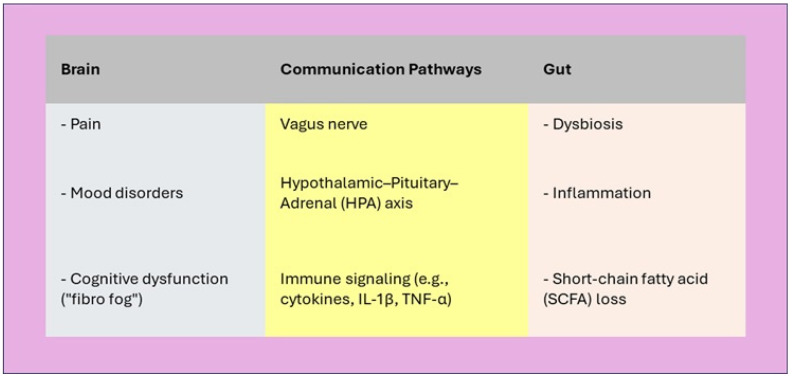
Gut–Brain Axis in Fibromyalgia. The gut–brain axis is dysregulated in fibromyalgia, contributing to pain amplification, mood disturbances, and cognitive deficits through immune, endocrine, and neural pathways.

Dysbiosis, or microbial imbalance, has been consistently reported in FM patients [60,61,62,63,64,65,66]. For example, a landmark study by Minerbi et al. (2019) reported significant alterations in the relative abundance of specific bacterial taxa in FM compared to healthy controls [67,68,69,70,71,72,73]. These microbial differences correlated with symptom severity and pain scores, suggesting a potential causal or modulatory role [74,75,76,77,78,79,80]. Gut microbiota may influence CNS activity through the production of Short-Chain Fatty Acids (SCFAs), tryptophan metabolism, neuroactive compound synthesis (e.g., GABA, serotonin precursors), and immune modulation [81,82,83,84,85,86,87]. These pathways are intimately involved in maintaining the integrity of the blood–brain barrier, modulating microglial activation, and influencing the release of pro-inflammatory cytokines such as IL-1β, IL-6, and TNF-α—molecules implicated in both mood disorders and chronic pain (Figure 2) [88,89,90,91,92,93,94].

Another layer of complexity is introduced by dietary habits, which are powerful modulators of microbial ecology [95,96,97,98,99]. Diets rich in fiber, polyphenols, and fermented foods can promote a more anti-inflammatory microbiota profile, while Western-style diets high in saturated fats and refined sugars may foster dysbiosis and systemic inflammation [100,101,102,103,104,105,106]. Nutritional interventions that target the microbiome may therefore hold promise as adjunctive strategies in FM [107,108,109,110,111].

### 1.3. Literature Gap: Need for Human Clinical Evidence

Although preclinical studies have enhanced understanding of gut–brain interactions, their translation to clinical practice remains limited. Much of the existing FM literature on microbiota modulation is theoretical or based on animal and small-scale human studies. Animal models have shown that microbiota interventions—such as antibiotics, probiotics, or fecal microbiota transplantation—can alter pain and stress responses, but human data are sparse. Systematic reviews on FM typically focus on pharmacological (e.g., antidepressants, anticonvulsants), behavioral (e.g., CBT, exercise), or nutritional supplements (e.g., magnesium, vitamin D), with limited attention to the gut microbiome. Existing reviews on microbiota-based interventions are often narrative or include non-human studies, limiting clinical relevance. To date, no comprehensive synthesis has examined human interventional trials targeting the microbiota in FM, particularly regarding cognitive or neuroinflammatory outcomes.

Given the complex nature of FM—spanning somatic, emotional, and cognitive domains—integrative treatment approaches that also target peripheral systems like the gut and immune axis are increasingly needed [112]. Due to the limited number of interventional studies conducted in FM populations, this review also includes related clinical cohorts exhibiting symptom or mechanistic overlap, such as chronic pain or mood disorders, to extract insights relevant to FM care.

### 1.4. Objective of the Systematic Review

This systematic review aims to synthesize clinical evidence from human studies evaluating microbiota-modulating interventions—such as probiotics, prebiotics, selective antibiotics (e.g., rifaximin), and dietary strategies—on FM-related outcomes. Specifically, the review will assess:

- pain outcomes, including self-reported pain intensity and pain thresholds;
- cognitive function, particularly attention, working memory, and mental fatigue;
- psychological and affective symptoms, including anxiety and depression;
- markers of systemic or neuroinflammation, such as hs-CRP, IL-1β, and TNF-α.

Secondary objectives include examining the impact of these interventions on quality of life, sleep, gut symptoms, and potential side effects or adherence issues [113,114,115,116,117,118,119]. Where available, the review will also explore associations between symptom improvement and microbiota composition or metabolomic changes, as assessed by fecal or blood biomarkers [120,121,122,123,124,125,126]. By focusing exclusively on human interventional studies (e.g., RCTs, open-label trials) and excluding in vitro and animal research, this review seeks to provide clinically applicable insights into the potential of microbiota-targeted therapies in FM. It may also help identify microbial targets for future interventions and subgroups of FM patients—such as those with gastrointestinal comorbidities or elevated inflammation—who could particularly benefit from such approaches [127,128,129,130,131,132,133].

## 2. Materials and Methods

### 2.1. Search Processing

The literature search was performed in accordance with the Preferred Reporting Items for Systematic Reviews and Meta-Analyses (PRISMA) guidelines. Three major databases—PubMed, Scopus, and Web of Science—were systematically searched using the following combination of keywords and MeSH terms: (“Neuroinflammation” OR “Fibromyalgia” OR “Nervous System Diseases”) AND (“Gastrointestinal Microbiome” OR “Microbiota” OR “Dysbiosis” OR “Probiotics”) AND (“Cognition” OR “Mood Disorders” OR “Depression” OR “Anxiety”). The search was limited to human studies published in English and covered the last 10 years, up to April 2025. This systematic review has been registered in the PROSPERO database (ID: 1064838) and is currently under review for formal approval. A total of 544 results were retrieved from PubMed, 478 from Scopus, and 303 from Web of Science, amounting to 1333 records. All search results were imported into a reference management software, and duplicates were removed. The remaining articles underwent an initial screening of titles and abstracts to assess relevance. Subsequently, full texts of potentially eligible studies were reviewed in detail. Studies were excluded if they were duplicates, not available as free full-text articles, conducted in vitro or on animal models, off-topic, or classified as reviews or meta-analyses. After applying these criteria, 11 articles met all inclusion criteria and were selected for the final analysis.

### 2.2. Inclusion and Exclusion Criteria

**Figure 3:**
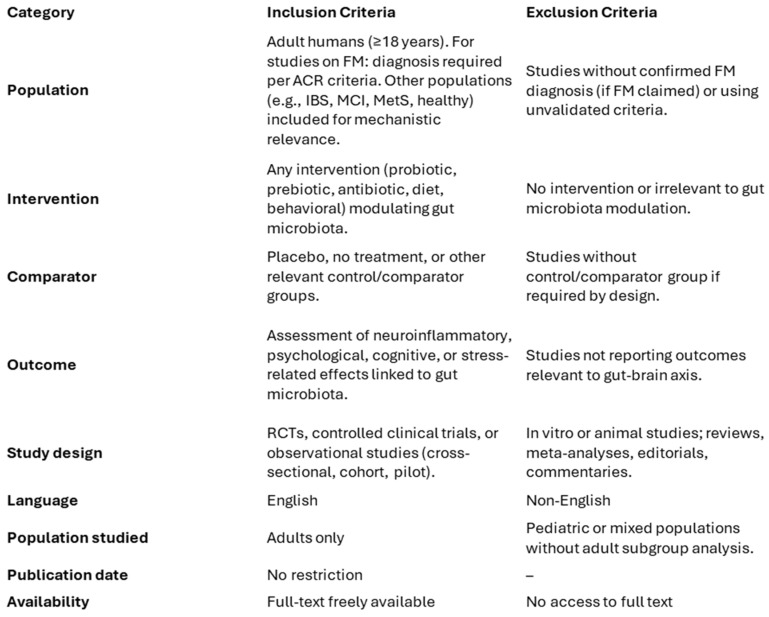
Summary of Inclusion and Exclusion Criteria.

Studies were selected according to predefined criteria aligned with PRISMA guidelines to ensure clarity and reproducibility. Eligible studies were original research articles published in English, with freely accessible full texts, conducted on adult human participants (≥18 years old). Both randomized controlled trials (RCTs), controlled clinical trials, and well-designed observational studies were included. The review considered studies investigating the relationship between gut microbiota and neuroinflammatory or neuropsychological outcomes, including cognitive impairment, stress reactivity, mood disorders, or pain perception. Studies were included regardless of participant clinical condition (e.g., fibromyalgia, metabolic syndrome, IBS, MCI, or healthy), as long as relevant mechanisms involving the gut–brain–immune axis were explored. However, for studies specifically involving fibromyalgia patients, only those with a confirmed diagnosis according to the American College of Rheumatology (ACR) criteria were included. Although a minimum follow-up of four weeks was prioritized, cross-sectional or mechanistic studies with shorter durations were considered if they contributed relevant insights into microbiota-related pathways. Exclusion criteria encompassed in vitro or animal models, reviews, meta-analyses, conference abstracts, editorials, or commentary pieces. Studies were excluded if they lacked a focus on psychological, cognitive, or inflammatory outcomes in relation to gut microbiota, or if fibromyalgia diagnosis was not supported by validated clinical criteria when relevant. A schematic summary of the inclusion and exclusion criteria is presented in Figure 3, in accordance with PRISMA best practices.

### 2.3. PICo Question

The systematic review was guided by the Population, Interest, and Context (PICo) framework. The population (P) comprised human subjects; the phenomenon of interest (I) was the gut microbiota; and the context (Co) involved neuroinflammatory processes and associated psychological outcomes. This framework facilitated the development of the search strategy and ensured a focused and relevant selection of studies addressing the central research question. The main outcomes evaluated across the included studies were: improvements in psychological well-being (e.g., reduced anxiety, stress, and depressive symptoms); enhancement of cognitive performance (e.g., attention, memory, concentration); reduction in systemic inflammation (e.g., lower levels of hs-CRP, IL-1β, TNF-α) and compositional and functional changes in the gut microbiota (e.g., increased SCFA-producing bacteria and microbial diversity).

These outcomes reflect the multifaceted impact of gut microbiota modulation on domains relevant to fibromyalgia and related conditions.

### 2.4. Data Processing

Data extraction was conducted using a pre-piloted spreadsheet in Excel, used by two independent reviewers. Extracted data included: author/year, study design, population characteristics, diagnostic criteria, intervention details, duration, outcome measures, analysis techniques (e.g., 16S rRNA), and key results. The ROBINS-I tool for non-randomized studies and the Cochrane RoB 2.0 tool for randomized controlled trials (RCTs) were used to evaluate the risk of bias. Key findings relevant to the link between gut microbiota and neuroinflammation were recorded. Any disagreements between reviewers were resolved through discussion and, when necessary, with input from a third reviewer. This process ensured the reliability and consistency of the data included in the final synthesis.

## 3. Results

### 3.1. Study Selection and Characteristics

**Figure 4:**
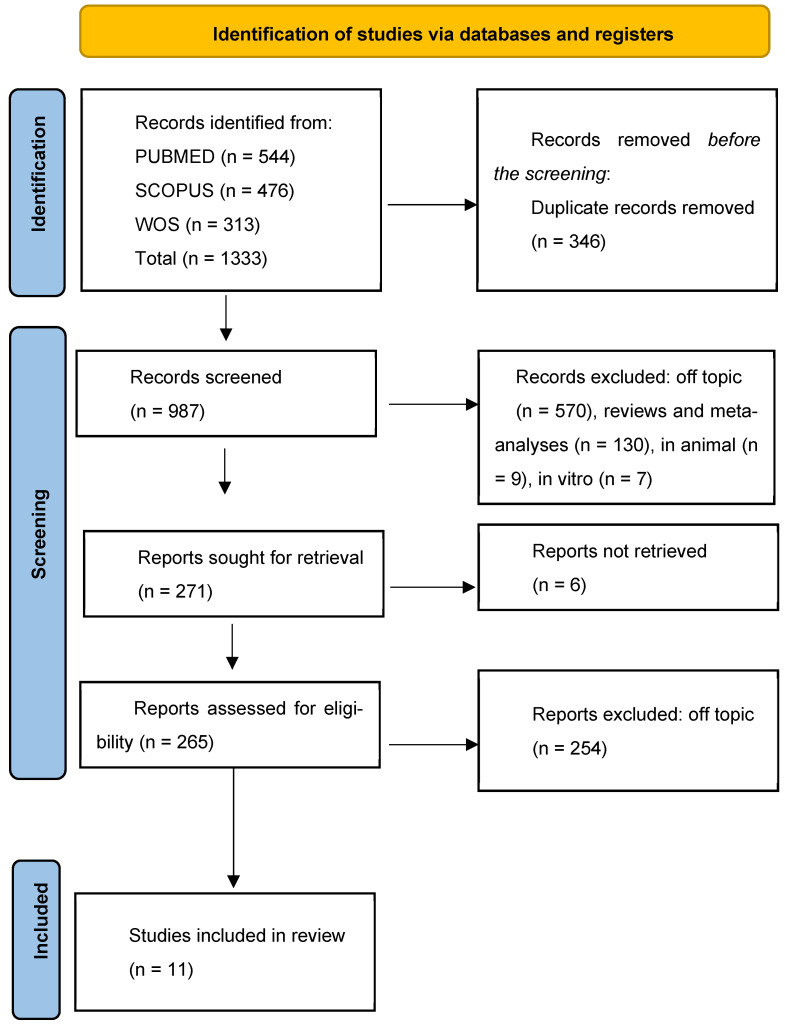
PRISMA Flow diagram of study selection.

The initial database search, conducted according to PRISMA guidelines, retrieved a total of 1333 records, 544 records from PubMed, 478 from Scopus, and 303 from Web of Science. After removing 346 duplicates, 987 records were assessed. Of these, 716 were excluded for being off-topic, reviews, meta-analyses, or non-human/in vitro studies. A total of 271 full-text articles were sought, of which 6 were not retrievable. Subsequently, 265 articles were reviewed for eligibility, and 254 were excluded for not meeting inclusion criteria, including lack of neuroinflammatory or psychological outcomes. Ultimately, 11 human studies, comprising randomized controlled trials and original research, were included in the qualitative synthesis. No quantitative synthesis (meta-analysis) was performed due to the high heterogeneity in study design, populations, interventions, and outcome measures, which precluded meaningful statistical pooling. The study selection process is illustrated in Figure 4, and the detailed characteristics of the included studies are summarized in Table 1.

### 3.2. Risk of Bias Assessment

The risk of bias was assessed using two validated tools: the Cochrane RoB 2 tool for randomized controlled trials (RCTs), and the ROBINS-I tool for non-randomized studies of interventions. For RCTs, seven key domains were considered, including potential bias from the randomization process, deviations from intended interventions, missing outcome data, measurement of outcomes, and selective reporting. Additionally, attention was given to declared conflicts of interest and the accuracy of microbiological methodologies used to assess the gut microbiota. For non-randomized studies, the ROBINS-I framework enabled a comprehensive evaluation of bias occurring before, during, and after the intervention, with a particular focus on the presence of confounding variables and selection biases.

Overall, most randomized studies demonstrated a low to moderate risk of bias in the main methodological areas. However, some concerns emerged in domains related to randomization procedures—especially in pilot trials with small sample sizes—and in the selective reporting of results, particularly in cases where study protocols had not been pre-registered. Non-randomized studies showed a generally higher risk of bias, which was most pronounced in relation to potential confounders and outcome measurement limitations, consistent with the inherent weaknesses of their designs.

**Figure 5:**
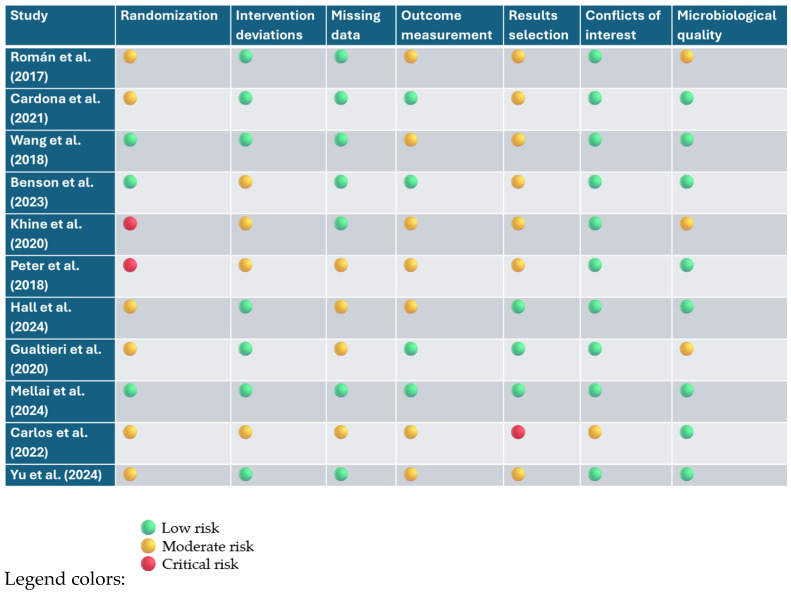
Risk assessment of bias (RoB 2.0 and ROBINS-I) [10,35,37,41,68,134,135,136,137,138,139].

The results of this evaluation are summarized in Figure 5. They support the overall reliability of the body of evidence reviewed, while also underscoring the need for future studies with more robust designs and greater methodological transparency. Notably, the studies by Khine and Peter exhibited the highest levels of bias among the included non-randomized studies [10,136]. Among the domains assessed, randomization procedures and selective outcome reporting were the most frequently problematic areas across trials. While the microbiological methods employed were generally appropriate, the level of detail provided was sometimes insufficient to allow for a full assessment of methodological rigor.

## 4. Discussion

### 4.1. Gut Microbiota in FM: A Growing Field of Interest

Recent studies have increasingly highlighted the gut microbiota’s involvement in systemic symptoms, mood disorders, and altered pain perception, underscoring its growing relevance in complex chronic conditions such as FM [140,141,142,143,144]. This multifactorial disorder, characterized by widespread musculoskeletal pain, fatigue, and neuropsychological dysfunction, is now being investigated through the lens of gut–brain–immune interactions [145,146,147,148,149].

### 4.2. Prebiotics and Immune-Mood Modulation

A notable contribution to this field is the pilot study by Hall et al., which evaluated the effects of prebiotic supplementation in individuals with metabolic syndrome (MetS), a condition often accompanied by low-grade systemic inflammation and psychological distress [137,150,151,152,153]. Over a 12-week intervention, participants receiving prebiotics exhibited a significant reduction in High-sensitivity C-reactive Protein (hs-CRP), a key biomarker of systemic inflammation. Additionally, marked improvements were reported in self-assessed stress, anxiety, and depressive symptoms [120,121,122,154,155]. However, these findings should be interpreted with caution due to the small sample size and the lack of microbiome sequencing data, which limit insights into mechanistic pathways. These findings suggest that modulating the gut microbiota through dietary fibers can influence not only immune function but also mental health, an effect likely mediated by the gut–brain axis, which may have clinical relevance in FM, where systemic inflammation and psychological symptoms often coexist. Although conducted in individuals with metabolic syndrome, the observed improvements in inflammation and psychological symptoms suggest gut-mediated mechanisms that may also be relevant to FM, where similar pathophysiological features are present.

### 4.3. IBS, Dysbiosis, and FM: Mechanistic Overlaps

Support for this hypothesis also comes from Peter et al., who analyzed the gut microbial composition in patients with IBS, identifying specific microbial signatures associated with psychological discomfort. Their study reinforces the emerging view that intestinal dysbiosis does not merely impact gastrointestinal function but plays a broader role in modulating emotional and cognitive states via neuroimmune pathways. These mechanisms are particularly relevant to FM, where chronic inflammation, psychological stress, and altered neuroimmune signaling are interwoven in symptom expression.

### 4.4. SCFA-Producing Bacteria and Neuroimmune Homeostasis

Further evidence for the microbiota’s therapeutic potential is provided by Mellai et al., who investigated the effects of Opuntia ficus-indica supplementation on gut microbiota in healthy subjects. Their results demonstrated an increased abundance of SCFA-producing bacteria, including *Bifidobacterium* and *Parabacteroides,* along with improvements in subjective well-being. Notably, these bacterial genera are known for their capacity to produce neuroactive metabolites like butyrate, which possess anti-inflammatory properties and are involved in supporting intestinal barrier integrity and central nervous system homeostasis.

### 4.5. Transgenerational Effects of Microbiota Modulation

The study by Yu et al. adds a transgenerational dimension to this discussion. In a cohort of mothers undergoing stress-reduction interventions during pregnancy, Yu and colleagues observed compositional changes in the maternal gut microbiota that were mirrored in their infants [136,156,157,158]. These alterations were accompanied by reductions in maternal stress levels, suggesting that psychosocial interventions can modulate gut microbial communities with downstream effects on offspring health.

### 4.6. Antibiotics, Brain Activity, and Social Stress

Expanding on this concept, Wang et al. explored the effects of short-term antibiotic administration on brain responses to social stress in healthy individuals. Participants treated with rifaximin, a non-absorbable antibiotic that alters gut microbiota, exhibited altered neural activity during a social exclusion task, particularly in prefrontal and cingulate regions. While the neuroimaging findings are compelling, the study’s short duration and small cohort limit its power to detect lasting clinical effects, particularly in patient populations. These changes were accompanied by a reduction in perceived social rejection, suggesting that gut microbiota modulation can influence emotional processing and stress reactivity.

### 4.7. Microglia, Inflammation, and Nociplastic Pain

Complementing these findings, Benson et al. provided a theoretical framework linking inflammation, mood disorders, and pain perception [134,159,160,161]. They emphasized how alterations in microbiota composition can modulate central nervous system processes, particularly through microglial activation and neurotransmitter imbalance, both of which are observed in FM [162,163,164,165,166].

### 4.8. Direct Evidence from FM Patients: Probiotic Interventions

Roman and Cardona investigated the clinical effects of multi-strain probiotics in FM patients. Their results showed a 15–20% reduction in pain scores compared to placebo, with parallel improvements in memory and concentration. As a randomized controlled trial in FM patients, this study provides stronger evidence, although the sample size was still limited, and microbiota changes were not directly analyzed. The response was particularly pronounced in patients with gastrointestinal comorbidities such as IBS, indicating that baseline microbiota profiles may influence therapeutic efficacy [167,168,169,170,171,172,173].

### 4.9. Host Genetics and Personalized Microbiota Therapies

Adding a genetic layer to this personalized approach, Gualtieri et al. examined the interaction between psychobiotic efficacy and host genetics. They found that individuals carrying the IL-1β A allele, a variant associated with hyperinflammatory responses, showed greater reductions in anxiety and IL-1β serum levels following probiotic intake [174,175,176,177]. Although promising, the study design was exploratory, and replication in larger, genetically stratified cohorts is needed.

### 4.10. Microbiota as Chronic Modulators of Inflammation

A more systemic perspective is offered by Carlos et al., who studied patients with schistosomiasis undergoing surgical correction of portal hypertension [137,178,179,180]. Despite anatomical resolution, persistent inflammation and fibrosis were observed postoperatively, pointing toward dysbiosis as a chronic driver of pathology [181,182,183,184,185]. This finding resonates with FM, where residual symptoms often persist despite standard care [186,187,188,189,190,191,192]. Even though the underlying disease is different, the persistent inflammation in the absence of active disease mirrors FM’s clinical trajectory, reinforcing the role of microbiota as a chronic modulator. This chronic inflammatory persistence despite resolution of primary insults mirrors the clinical trajectory seen in FM, reinforcing the need to consider microbiota as a long-term modulator rather than a transient player [138,193,194,195,196].

### 4.11. Mindfulness, SCFAs, and Cognitive Improvement

Finally, Khine et al. explored non-pharmacological strategies by analyzing the effects of mindfulness practices in patients with mild cognitive impairment. Their work linked these interventions to an increase in SCFA-producing bacteria and improvements in cognitive performance, likely via modulation of the hypothalamic–pituitary–adrenal (HPA) axis and enhancement of gut barrier function. Such approaches may be particularly valuable in FM, where cognitive symptoms (“fibro fog”) are prominent and pharmacological treatments often yield suboptimal results.

### 4.12. Synthesis of Evidence and Future Directions

**Figure 6:**
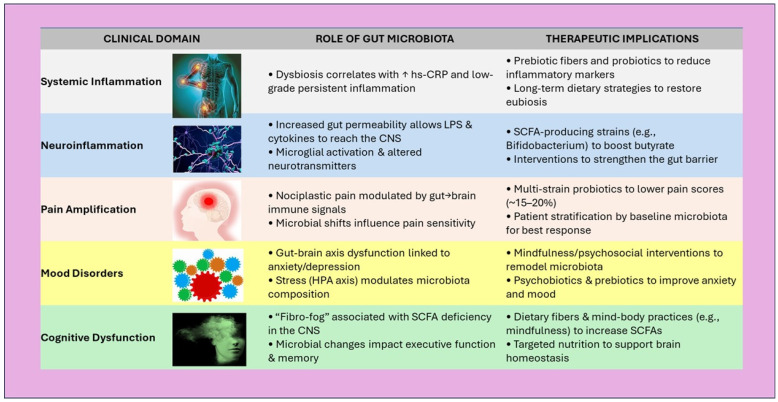
Schematic overview of clinical domains in FM, illustrating gut microbiota contributions and potential interventional strategies. ↑ = improvement/increase. Abbreviations: SCFA—Short-Chain Fatty Acids, hs-CRP—High-Sensitivity C-Reactive Protein, LPS—Lipopolysaccharide, CNS—Central Nervous System.

In conclusion, the integrative perspective emerging from these eleven studies positions the gut microbiota as a central modulator of inflammation, mood, and pain, all key dimensions of FM [139,197,198,199]. The cumulative evidence suggests that targeted interventions, ranging from prebiotics and probiotics to stress reduction and lifestyle modifications, could offer multi-systemic benefits. However, several methodological limitations must be acknowledged, including small, heterogeneous samples, diverse intervention protocols, and variability in outcome measures [200,201,202,203,204,205,206]. This heterogeneity complicates direct comparisons and limits the generalizability of findings. Additionally, many of the included studies are preliminary or exploratory in nature, lacking long-term follow-up or placebo-controlled designs [207,208,209,210,211,212,213]. These factors should temper the interpretation of results and underscore the need for more standardized, large-scale investigations. Figure 6 summarizes the proposed mechanisms linking gut microbiota to key FM symptom domains, offering a visual framework for potential intervention points.

Although this review primarily focuses on FMS and related neuroinflammatory conditions, several studies involving non-FMS populations (e.g., healthy individuals, patients with metabolic syndrome, irritable bowel syndrome, or mild cognitive impairment) were included due to overlapping pathophysiological mechanisms. Symptoms such as anxiety, visceral pain, cognitive impairment, and immune dysregulation strongly align with FM features, offering biologically plausible insights.

### 4.13. Toward Personalized, Microbiota-Based Therapies in FM

**Figure 7:**
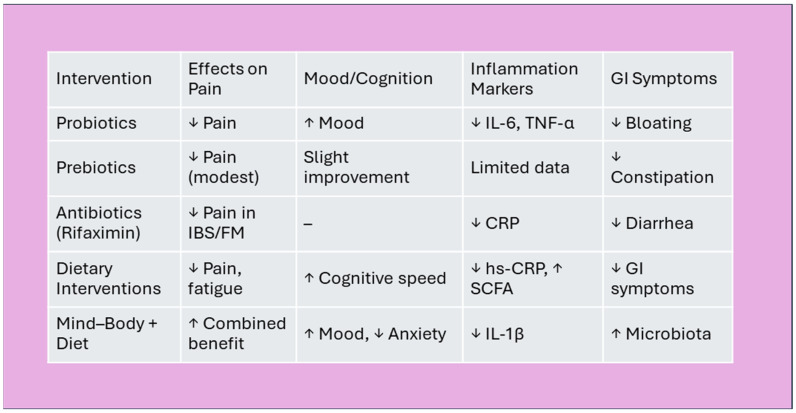
Summary of microbiota-based interventions. Effects of microbiota interventions on FM symptoms. Legend: ↓ = reduction; ↑ = improvement/increase; “–” = no significant change. Abbreviations: IBS—Irritable Bowel Syndrome, FM—Fibromyalgia, CRP—C-reactive Protein, hs-CRP—High-sensitivity C-reactive Protein, SCFA—Short-Chain Fatty, IL-6—Interleukin-6, TNF-α—Tumor Necrosis Factor-alpha, IL-1β—Interleukin-1 beta.

Future research should aim to delineate microbial signatures specific to FM and evaluate long-term outcomes through robust, longitudinal designs. Personalized strategies that integrate microbiota profiling, host genetics, and psychosocial context may ultimately redefine therapeutic paradigms for FM and related disorders (Figure 7) [67,135,214,215,216].

## 5. Limitations of the Included Studies

Despite the promising findings highlighted in this review, several limitations must be acknowledged regarding the included studies. First, many of the trials were pilot or exploratory in nature, with small sample sizes that reduce statistical power and generalizability. Second, there was considerable heterogeneity in study design, population characteristics, intervention protocols (e.g., strain types, dosages, duration), and outcome measures, which precluded meaningful meta-analysis and limited direct comparisons across studies. Third, the majority of studies lacked long-term follow-up, making it difficult to assess the durability of observed effects. Moreover, in several cases, gut microbiota composition was inferred from limited sampling or reported without detailed taxonomic resolution, and microbiome sequencing techniques were inconsistently applied. Some studies also presented a moderate to high risk of bias, particularly in relation to randomization procedures, lack of blinding, or selective reporting. Lastly, only a few trials specifically targeted patients with fibromyalgia, while others included broader populations with symptom or mechanistic overlap, potentially diluting the specificity of the findings for FM. These limitations underscore the need for future high-quality, FM-specific clinical trials with standardized methodologies and rigorous reporting.

## 6. Conclusions

The current evidence suggests that modulation of the gut microbiota may influence key pathophysiological mechanisms relevant to fibromyalgia, particularly neuroinflammation, stress reactivity, and cognitive-emotional dysregulation. Although only a minority of the included studies directly investigated FM populations, consistent findings across diverse clinical contexts—such as improvements in attention, reductions in perceived stress, and shifts in microbial composition—support the hypothesis that targeting the gut–brain axis can impact symptom domains commonly affected in FM. Notably, several interventions, including probiotics and prebiotics, were associated with increased abundance of short-chain fatty acid–producing bacteria and reductions in inflammatory markers, highlighting potential biological pathways of relevance. However, due to the predominance of small, exploratory trials and the heterogeneity of study populations and methodologies, these findings remain preliminary. Robust, FM-specific clinical trials are required to validate the therapeutic relevance of microbiota-targeted strategies and to clarify their role within integrated, personalized treatment models.

## Figures and Tables

**Table 1:** Summary of selected studies.

Authors	Type of Study	Aim of the Study	Materials and Methods	Main Outcomes	Results
Roman et al. (2017) [37]	Double-blind RCT, pilot study	To evaluate whether an 8-week probiotic regimen can improve physical, emotional, and cognitive symptoms in fibromyalgia syndrome (FMS) patients	60 FMS patients randomized to probiotics vs. placebo for 8 weeks. Tools: validated questionnaires (pain, mood), cognitive tasks (E-Prime), biological markers (urine cortisol, fecal microbiota).	Pain, mood, cognition, cortisol, microbiota	Improved emotional symptoms and cognitive performance in probiotic group (*p* < 0.05). No significant effect on pain. Decreased cortisol and increased microbiota diversity.
Cardona et al. (2021) [68]	Randomized controlled trial (RCT), pilot study	To assess the effects of multispecies probiotics on cognitive performance in FMS patients	31 FMS patients randomized to probiotics or placebo for 8 weeks. Cognitive tasks focused on memory and attention, pre- and post-treatment.	Attention, memory	Improved attention (reduced omission errors in Go/No-Go task; *p* < 0.05) in probiotic group. No significant change in memory.
Wang et al. (2018) [134]	Double-blind RCT	To examine the effect of rifaximin on neural responses to social stress in healthy individuals	16 healthy adults randomized to rifaximin or placebo for 7 days. Brain activity assessed by MEG at rest and during a social exclusion task (Cyberball).	Stress perception, brain activity	Rifaximin group showed increased resting-state alpha power (prefrontal/cingulate cortex) and modulated beta power during social stress. Lower perceived exclusion reported (*p* < 0.05).
Benson et al. (2023) [135]	Double-blind, placebo-controlled crossover fMRI study	To assess the interaction between inflammation and mood on visceral pain perception	39 healthy volunteers received LPS or saline, underwent pain stimulation under sad vs. neutral mood. Brain activity measured with fMRI.	Pain sensitivity, brain response	Both LPS and sad mood increased pain unpleasantness and activation in striatal/limbic areas. Their combination intensified effects, highlighting interaction between mood and inflammation.
Khine et al. (2020) [10]	Randomized controlled trial (RCT)	To investigate if mindfulness training affects gut microbiota in mild cognitive impairment (MCI)	Older adults with MCI received mindfulness intervention; cognitive testing, inflammatory biomarkers, and microbiota analysis were conducted pre- and post-treatment.	Cognition, inflammation, microbiota	Improved cognitive performance correlated with specific changes in gut microbiota. No significant systemic inflammation change.
Peter et al. (2018) [136]	Observational cohort study with machine learning	To identify gut microbial features associated with psychological distress in IBS patients	48 IBS patients underwent psychological evaluation and microbiota profiling (16S rRNA). Data analyzed with ML, diversity indices, and correlation.	Anxiety, depression, microbiota composition	Psychological distress was linked to distinct microbial patterns (↓ Lachnospiraceae in depression, ↑ Proteobacteria/Bacteroidaceae in anxiety). ML classified profiles with high accuracy.
Hall et al. (2024) [137]	Pilot open-label RCT	To examine effects of prebiotic fiber on inflammation, mood, and microbiota in metabolic syndrome (MetS)	60 MetS patients randomized to prebiotic + diet vs. diet only for 12 weeks. Mood scales, CRP, and fecal microbiota (16S) assessed.	Mood, inflammation, microbiota	Prebiotic group showed reduced hs-CRP and improved mood scores (*p* < 0.01). Increased SCFA-producing bacteria observed.
Gualtieri et al. (2020) [41]	Randomized, placebo-controlled RCT	To assess probiotics’ effect on anxiety, especially in IL-1β polymorphism carriers	150 adults randomized to receive probiotics or placebo for 12 weeks. Psychological questionnaires and genetic analysis conducted.	Anxiety, genetic susceptibility	Significant reduction in anxiety in the probiotic group, especially among IL-1β A allele carriers (*p* < 0.05).
Mellai et al. (2024) [138]	Randomized, double-blind, placebo-controlled RCT	To evaluate efficacy of Opuntia ficus-indica extract on gut health in dysbiosis	80 adults with dysbiosis randomized to Odilia™ or placebo for 8 weeks. Microbiota (16S), GIQLI, and GSAS used as outcome measures.	GI symptoms, microbiota composition	Odilia™ improved microbiota profile (↑ Bacteroides, ↓ Firmicutes/Bacteroidetes ratio), reduced inflammation, and enhanced GI symptom scores.
Carlos et al. (2022) [35]	Randomized, double-blind, placebo-controlled RCT	To assess whether probiotics reduce binge eating and food addiction after bariatric surgery	101 patients post-gastric bypass received probiotics or placebo for 90 days, followed up to 1 year. Assessed with YFAS and BES.	Food addiction, binge eating	Probiotic group maintained reduction in binge eating and food addiction scores at 1-year follow-up. Placebo group relapsed.
Yu et al. (2024) [139]	Randomized controlled trial (RCT), secondary analysis	To assess whether maternal stress reduction alters maternal and infant microbiomes	38 mother–infant pairs randomized to relaxation training or control. Fecal and milk microbiomes assessed at baseline and 8 weeks.	Microbiota (maternal, milk, infant)	Relaxation training modified microbiomes in maternal gut, breast milk, and infant gut. Increased Bifidobacterium in milk and greater infant microbial diversity observed.

↑—increase; ↓—decrease. Abbreviations. BES—Binge Eating Scale; CRP—C-reactive Protein; E-Prime—E-Prime software for psychological experiments; fMRI—Functional Magnetic Resonance Imaging; FMS—fibromyalgia syndrome; GI—Gastrointestinal; GIQLI—Gastrointestinal Quality of Life Index; GSAS—Gastrointestinal Symptom Assessment Scale; hs-CRP—High-sensitivity C-reactive Protein; IBS—irritable bowel syndrome; IL-1β—Interleukin 1 beta; LPS—Lipopolysaccharide; MCI—mild cognitive impairment; MEG—Magnetoencephalography; MetS—metabolic syndrome; ML—machine learning; RCT: randomized controlled trial; SCFA—Short-Chain Fatty Acids; YFAS—Yale Food Addiction Scale.

## Abbreviations

BES	Binge Eating Scale
CFS	Chronic Fatigue Syndrome
CFU	Colony Forming Units
CNS	Central Nervous System
CRP	C-Reactive Protein
DMN	Default Mode Network (brain functional network)
E-Prime	E-Prime Software for Psychological Experiments
FM	Fibromyalgia
FMS	Fibromyalgia Syndrome
FMT	Fecal Microbiota Transplantation
FOS	Fructooligosaccharides (a type of prebiotic fiber)
GABA	Gamma-Aminobutyric Acid
GI	Gastrointestinal
GIQLI	Gastrointestinal Quality of Life Index
GSAS	Gastrointestinal Symptom Assessment Scale
HADS	Hospital Anxiety and Depression Scale
HE	Hepatic Encephalopathy
HPA	Hypothalamic–Pituitary–Adrenal
hs-CRP	High-Sensitivity C-Reactive Protein (marker of systemic inflammation)
IBS	Irritable Bowel Syndrome
IL-1β	Interleukin 1 Beta (Proinflammatory Cytokine)
LPS	Lipopolysaccharide
MCI	Mild Cognitive Impairment
MEG	Magnetoencephalography
MetS	Metabolic Syndrome
ML	Machine Learning
RCT	Randomized Controlled Trial
SCFA	Short-Chain Fatty Acids
SCFAs	Short-Chain Fatty Acids
YFAS	Yale Food Addiction Scale
fMRI	Functional Magnetic Resonance Imaging
